# MRI of female genital tract congenital anomalies: European Society of Urogenital Radiology (ESUR) guidelines

**DOI:** 10.1007/s00330-020-06750-8

**Published:** 2020-03-27

**Authors:** Cristina Maciel, Nishat Bharwani, Rahel A. Kubik-Huch, Lucia Manganaro, Milagros Otero-Garcia, Stephanie Nougaret, Celine D. Alt, Teresa Margarida Cunha, Rosemarie Forstner

**Affiliations:** 1 Serviço de Imagiologia, CHVNG/E, Rua Conceição Fernandes, 4434-502 Vila Nova de Gaia, Portugal; 2 Departamento de Medicina, Faculdade de Medicina, Universidade do Porto, Alameda Prof. Hernâni Monteiro, 4200-319 Porto, Portugal; 3 Department of Radiology, St Mary’s Hospital, Imperial College Healthcare NHS Trust, Praed Street, London, W2 1 NY UK; 4 Department of Surgery and Cancer, Imperial College London, London, UK; 5 Institut für Radiologie, Kantonsspital Baden AG, CH-5404 Baden-Dättwil, Switzerland; 6 Dipartimento di Scienze Radiologiche, Oncologiche e Anatomo Patologiche, Sapienza Università di Roma, Vle Regina Elena 324, 00161 Rome, Italy; 7 Department of Radiology, Hospital Universitario de Vigo, Planta 3 Vela A, Vigo, Spain; 8 Department of Radiology, Montpellier Cancer institute, INSERM, U1194, University of Montpellier, 208 Ave des Apothicaires, 34295 Montpellier, France; 9 Department of Diagnostic and Interventional Radiology, Medical Faculty, University Dusseldorf, Duesseldorf, Germany; 10 Serviço de Radiologia, Instituto Português de Oncologia de Lisboa Francisco Gentil, Rua Prof. Lima Basto, 1099-023 Lisbon, Portugal; 11 Department of Radiology, Universitätsklinikum Salzburg, PMU, Müllner-Hauptstr. 48, A-5020 Salzburg, Austria

**Keywords:** Genitalia, female, Guideline, Magnetic resonance imaging, Müllerian duct, Classification

## Abstract

**Objective:**

To develop imaging guidelines for the MR work-up of female genital tract congenital anomalies (FGTCA).

**Methods:**

These guidelines were prepared based on a questionnaire sent to all members of the European Society of Urogenital Radiology (ESUR) Female Pelvic Imaging Working Group (FPI-WG), critical review of the literature and expert consensus decision.

**Results:**

The returned questionnaires from 17 different institutions have shown reasonable homogeneity of practice. Recommendations with focus on patient preparation and MR protocol are proposed, as these are key to optimised examinations. Details on MR sequences and planning of uterus-orientated sequences are provided.

**Conclusions:**

The multiplanar capabilities and soft tissue resolution of MRI provide superb characterisation of the wide spectrum of findings in FGTCA. A standardised imaging protocol and method of reporting ensures that the salient features are recognised, contributing to a correct diagnosis and classification of FGTCA, associated anomalies and complications. These imaging guidelines are based on current practice among expert radiologists in the field and incorporate up to date information regarding MR protocols and essentials of recently published classification systems.

**Key Points:**

*• MRI allows comprehensive evaluation of female genital tract congenital anomalies, in a single examination.*

*• A dedicated MRI protocol comprises uterus-orientated sequences and vaginal and renal evaluation.*

*• Integration of classification systems and structured reporting helps in successful communication of the imaging findings.*

**Electronic supplementary material:**

The online version of this article (10.1007/s00330-020-06750-8) contains supplementary material, which is available to authorized users.

## Introduction

The majority of female genital tract congenital anomalies (FGTCA) affect the uterus. However, the spectrum of FGTCA is large, encompassing anomalies of the cervix, vagina, vulvar introitus and fallopian tubes, with or without associated malformations of the ovary, urinary tract, skeleton and other organs [1, 2].

Although the embryology of the female genital tract is complex and still not fully understood, a basic knowledge is important for a better understanding of the pathogenesis of FGTCA and associated anomalies [3].

FGTCA can be comprehensively evaluated with magnetic resonance imaging (MRI) to aid in characterisation of the anomaly and assessment of associated complications and for preoperative planning [4–7].

Several classification systems have been proposed to describe FGTCA. The most recent was published in 2013 by the European Society of Human Reproduction and Embryology/European Society for Gynaecological Endoscopy (ESHRE/ESGE) [8].

This paper will provide guidance on patient preparation, dedicated MRI protocols and a structured report format to allow for uniform state-of-the-art examination of FGTCA.

## Materials and methods

These guidelines were prepared by the Female Pelvic Imaging Working Group (FPI-WG) of the European Society of Urogenital Radiology (ESUR), based on a questionnaire sent to all FPI-WG members, critical review of the literature and expert consensus decision.

### Literature review

Published literature was collected through a Medline search of abstracts, from 1 January 2004 to 31 January 2019, including the keywords: ‘Mülllerian duct anomalies’ and ‘MR imaging’.

The results were limited to English language, to full text available and to publications from important radiology, gynaecology and urology journals (impact factor above 1).

Articles were organised in main topics and shared electronically with all authors via Dropbox® (Dropbox Inc.).

### Questionnaire

A questionnaire was proposed (C.M.) and then refined by two further authors (N.B., R.F.). The topics addressed include indications, clinical practice, patient preparation, MRI protocol and reporting (including use of classification systems and structured reports). The role of computed tomography (CT) and the clinical practice of 2D/3D gynaecological ultrasound (US) were also assessed.

### Data extraction and consensus meeting

ESUR FPI-WG members were invited via email to complete the questionnaire based on local practice. Returned questionnaires were reviewed and data extracted (C.M.). Results were presented and discussed at the FPI-WG meeting at ECR 2019 in Vienna, Austria.

After the meeting, preliminary patient preparation and a detailed MR protocol were developed by the lead authors (C.M., N.B., R.F.). This was sent via email to all those involved in the guideline. Contributions were incorporated and a final version of MR protocol and patient preparation was reached.

Consensus was defined as at least 80% agreement among experts.

## Results

### Participating institutions

Twenty questionnaires were returned from 17 different institutions: 18 from Europe, 1 from Japan and 1 from the USA:

- Portugal: *n* = 4 (2 from the same institution, same protocol)
- Serbia: *n* = 3 (from the same institution, same protocol)
- Italy: *n* = 2
- Greece: *n* = 2
- UK: *n* = 2
- Austria: *n* = 1
- France: *n* = 1
- Switzerland: *n* = 1
- Spain: *n* = 1
- Germany: *n* = 1
- Japan: *n* = 1
- USA: *n* = 1

The return rate according to the total number of FPI-WG members (*n* = 57) was 35%. Several FPI-WG members were unable to collaborate, as their practice is mainly oncological imaging, with little or no congenital anomaly work.

### MRI exams reported per year

Among respondents, the approximate number of MRI exams performed and/or reviewed per year for assessing FGTCA is the following:

- Less than 10 exams: *n* = 7
- 10–20 exams: *n* = 3
- 20–50 exams: *n* = 6
- More than 50 exams: *n* = 1

### Indications for MR imaging of FGTCA

The most common indications for MRI in FGTCA are to clarify imaging findings at gynaecological US (*n* = 16), hysterosalpingogram (*n* = 12) or CT (*n* = 10) (Table 1).

**Table 1:** Indications for MRI in patients with FGTCA in order of frequency

• Indeterminate or other unclear findings at gynaecological US	*n* = 16/17
• Primary amenorrhea	*n* = 14/17
• Infertility work-up	*n* = 13/17
• Known FGTCA, MRI for treatment planning	*n* = 13/17
• Suspected FGTCA at hysterosalpingogram	*n* = 12/17
• Pelvic pain, dysmenorrhea, dyspareunia, other clinical symptoms	*n* = 10/17
• Suspected FGTCA at CT	*n* = 10/17
• Monitoring after treatment	*n* = 8/17
• Other*	*n* = 3/17

*To differentiate FGTCA from adnexal masses, particularly in combination with endometriosis; to illustrate more in detail findings of sonography by gynaecologists; suspected anomaly because of abnormal external genitalia on physical exam

### Role of computed tomography

**Fig. 1:**
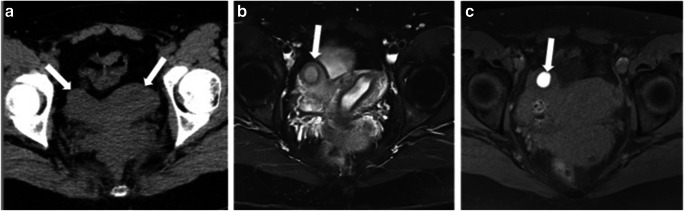
Role of CT in FGTCA assessment. **a** Non-reported FGTCA in CT examination performed for renal colic. Unenhanced CT shows an abnormal uterine contour (arrows), suggesting a uterine malformation. The patient returned some years later for pelvic MRI in the context of infertility work-up. T2W FS (**b**) shows a right rudimentary horn (arrow) not communicating with the main uterine cavity. T1W FS (**c**) depicts high SI in the rudimentary cavity, in keeping with haematometra (arrow). It is important to keep a high grade of suspicion, as early diagnosis of FGTCA may help to avoid prolonged symptomatic periods and the complications that may subsequently arise, such as infertility and endometriosis

The majority of respondents (82%) did not see any role for CT in assessing FGTCA. Three believe that CT can have a role in selected cases: complementary to MRI to characterise associated renal anomalies or other anomalies in the abdomen, and occasionally as the first imaging modality to suggest the presence of a FGTCA, when the CT is performed for another reason (Fig. 1).

### 3D ultrasound

None of the responding radiologists performs 3D US for FGTCA themselves; however, it is performed by gynaecologists in several institutions. Only one respondent reported access to 3D US for correlation with MRI.

### Patient preparation

In most institutions (*n* = 15/17; 88% agreement - consensus), the patient is not scheduled according to the *menstrual cycle*. In the remaining 2 institutions, patients are scheduled in the secretory phase or ‘mid-cycle’.

The majority of respondents (*n* = 14/17; 83% agreement - consensus) ask the patient *clinical questions* or ask them to complete a questionnaire before the exam (e.g. time of last menstruation, clinical symptoms, previous gynaecological surgery).

The majority of the institutions (*n* = 13/17; 77% agreement - no consensus) ask the patient to *fast* before examinations to reduce artefact from bowel motion. Duration of fast ranges between 2 and 6 h, mostly 4 h (*n* = 7).

In most institutions (*n* = 15/17; 88% agreement - consensus), *spasmolytic agents* are used on a regular basis. Butylscopolamine was the most commonly used (*n* = 10), while glucagon was used in three institutions (*n* = 3). In the four remaining institutions, both spasmolytic agents were available. Butylscopolamine was used by intravenous (IV) administration in 6 institutions and by intramuscular (IM) administration in other 6 institutions. Glucagon was used by IV administration in 3 institutions and by IM administration in 4 institutions.

Patients should achieve a moderately distended *urinary bladder*. This is usually achieved by instructing the patient to empty their bladder 1 h prior to the examination (*n* = 17/17; 100% agreement - consensus).

In 9 institutions, vaginal opacification with US gel is performed (*n* = 9/17, 53% agreement), routinely (*n* = 3/17) or in selected cases (*n* = 6/17). In 8 institutions, vaginal opacification is not performed (no consensus).

Gel instillation was performed by a nurse, radiology technician, radiologist or the patient themselves, according to the local department resources and practices. Indications for vaginal opacification are listed (Table 2).

**Table 2:** Indications for vaginal opacification with US gel

• Assessment of vaginal and cervical morphology	
• Suspicion of uterine agenesis	
• Suspicion of vaginal septum	
• Visualisation of vaginal atresia/partial aplasia	
• Characterisation of vaginal septum (length, thickness)	
• Evaluation of vaginal length in partial agenesis	

### Patient positioning

Patient positioning is not uniform: supine and head first (*n* = 8/17) and supine and feet first (*n* = 9/17). In one institution, head first is routinely used and feet first in cases of severe claustrophobia.

### MR equipment

Different MRI scanners (Philips, GE, Siemens) are used, with 1.5-T and 3.0-T magnetic field strength.

In 9 institutions, both 1.5-T and 3.0-T platforms were available. 3.0 T was preferred over 1.5 T in 7 cases; in one case, 1.5 T was preferred over 3.0 T.

A variety of coils are used for imaging, with a preference for pelvic phased array and multi-channel body coils.

### MR protocol

Imaging of kidneys is performed routinely in all except one institution (*n* = 16/17; 94% agreement) using T2 HASTE (coronal (*n* = 7), axial (*n* = 4) or both planes (*n* = 5)).

All protocols include T1W imaging (*n* = 17/17; 100% agreement). The majority of institutions (*n* = 14/17 83% agreement) perform T1W imaging without and with fat suppression (FS). T1W without FS only is performed in 3 institutions. T1W sequences used were standard sequences and/or Dixon technique. Overall, Dixon technique is used in 8 institutions. Axial plane was used in all institutions, with one adding the sagittal plane.

All institutions performed multiplanar T2W sequences of the pelvis in strictly axial, sagittal and/or coronal orientation (*n* = 17/17, 100% agreement), in two (*n* = 7/17) or three planes (*n* = 7/17), with only one plane in three institutions (*n* = 3/17). All except one institution also performed uterus-orientated T2W sequences (*n* = 16/17, 94% agreement).

T2W uterus-orientated sequences most commonly obtained were a combination of true coronal and true axial of the uterus fundus and body (*n* = 13/17, 76% agreement). T2W true axial of the cervix was obtained in 7 institutions (*n* = 7/17, 42% agreement).

One institution obtains T2W parasagittal sequence, planned to get in the same alignment of the uterus and the cervix, whenever there is a significant lateral deviation of uterine body and fundus relative to the cervix.

3D-T2W sequence of the pelvis, allowing multiplanar reformatting (MPR) of the uterus, is used in 2 institutions. A further 2 respondents remarked that they have trialled this sequence but found image quality to be low.

Three institutions use IV gadolinium for characterisation of FGTCA routinely, 8 depending on the clinical question and findings on pre-contrast sequences and the remaining 6 institutions do not use gadolinium. Indications for IV gadolinium include evaluation of concomitant findings (ovarian or myometrial masses, endometrial polyps), suspicion of abscess/infection or after treatment (e.g. metroplasty). MR-urography is added in cases of urinary tract obstruction at one institution.

### Reporting

Standardised proforma reporting is only performed at 3 institutions (*n* = 3/17).

In the MRI report, all (*n* = 17/17) include the following: size and morphology of the uterus, cervix and ovaries, ovarian position and associated findings (such as endometriosis, adhesions, and bone anomalies). Almost all (*n* = 15/17) give details on the vagina (size, morphology) and kidneys.

Other information that might be included is clinical information from the questionnaire, pelvic fluid, lymph nodes and any other anomaly detected (in liver, bowel).

Almost all respondents apply a classification system for FGTCA (*n* = 15/17), with distribution detailed in Supplementary Material Table 1.

### Clinical meeting

Six institutions discuss the imaging of FGTCA cases in multidisciplinary meetings.

## Discussion

### Patient preparation

Patient preparation is summarised in Table 3.

**Table 3:** Patient preparation

Exam schedule	Scheduling the exam according to the menstrual cycle is not necessary
Clinical questions	Clinical questions should be asked before the exam (e.g. time of last menstruation, clinical symptoms, hormonal medication, prior surgical procedures)
Fasting*	Fasting before the exam may be useful (3–6 h)
Bladder	Patients should empty bladder 1 h prior to examination in order to achieve a moderately filled bladder during the scan
Antiperistaltic agents	The use of antiperistaltic agents is recommended (20 mg butylscopolamine IV/IM or 1 mg of glucagon IV) unless contraindicated
Vaginal gel*	The use of vaginal gel may be useful, whenever feasible; ± 60 mL. Self-administration is an option

*No consensus was reached

#### Scheduling

Scheduling of MRI examination according to the menstrual cycle is not necessary. This agrees with the majority of authors in the literature [9].

#### Clinical questionnaire

It is recommended to obtain specific clinical information at the time of the scan: date of last menstruation, clinical symptoms and previous gynaecological surgery.

#### Fasting and spasmolytic agents

In line with previous ESUR guidelines [9–11], it is recommended to fast before the examination (3–6 h) and to administer spasmolytic agents (20 mg of butylscopolamine or 1 mg glucagon (IV/IM), unless contraindicated) to reduce bowel motion artefact and obtain high-quality MR imaging.

#### Bladder

It is recommended to achieve a moderately filled bladder, in line with previous ESUR guidelines [9–11].

#### Vaginal gel

**Fig. 2:**
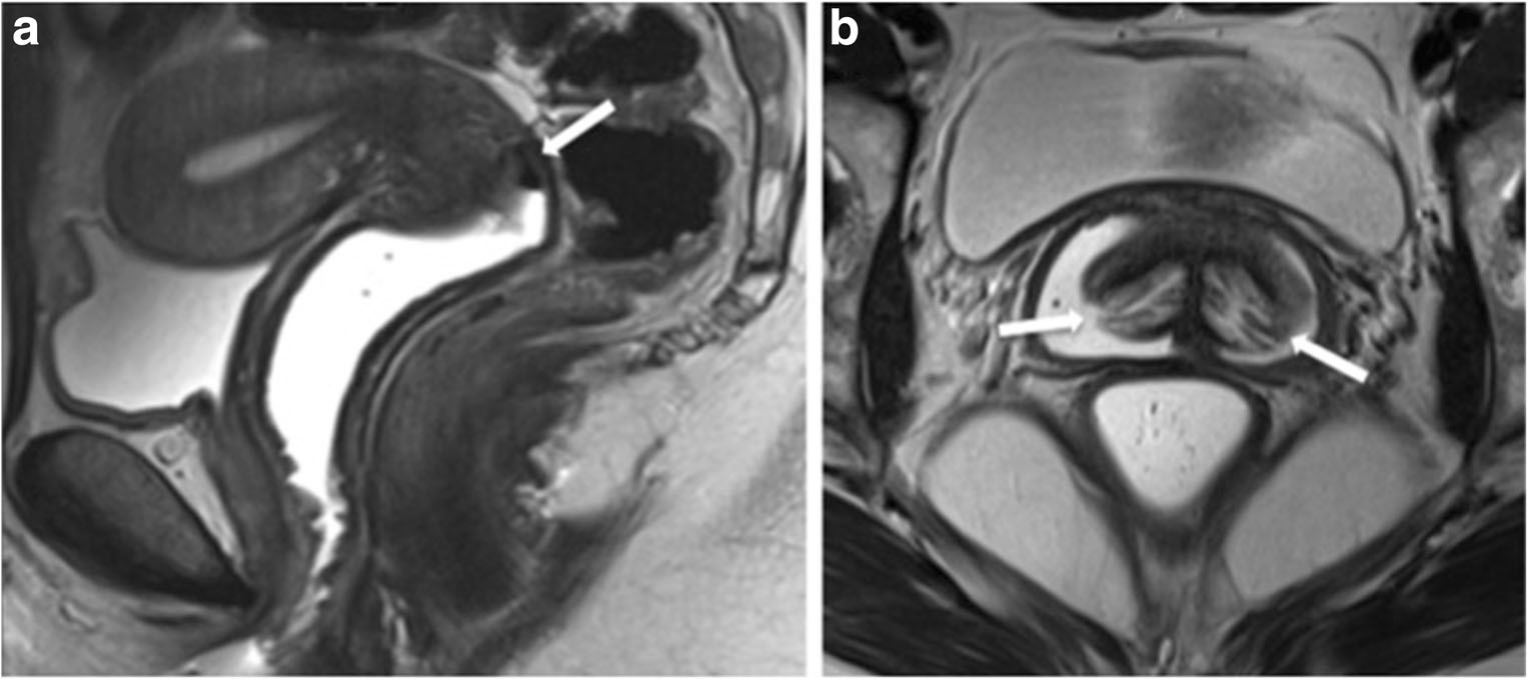
Patient preparation–vaginal opacification. **a** Self-application of US gel, about 60 mL, in the locker room, just before going to the MRI scanner room. T2W TSE sagittal image (**a**) showing well-distended vagina, allowing for optimal evaluation. A small amount of air is seen in the posterior vaginal fornix (arrow). The high T2 SI of the gel offers a good contrast with the vaginal walls or a septum (both low SI). **b** In another patient, with septate uterus with double cervix (arrows), the vaginal gel allows for optimal assessment of the cervical morphology

Vaginal opacification with gel can be useful in the assessment of vaginal anomalies, which are often overlooked in the absence of appropriate vaginal distension (Fig. 2a) [12, 13].

**Fig. 3:**
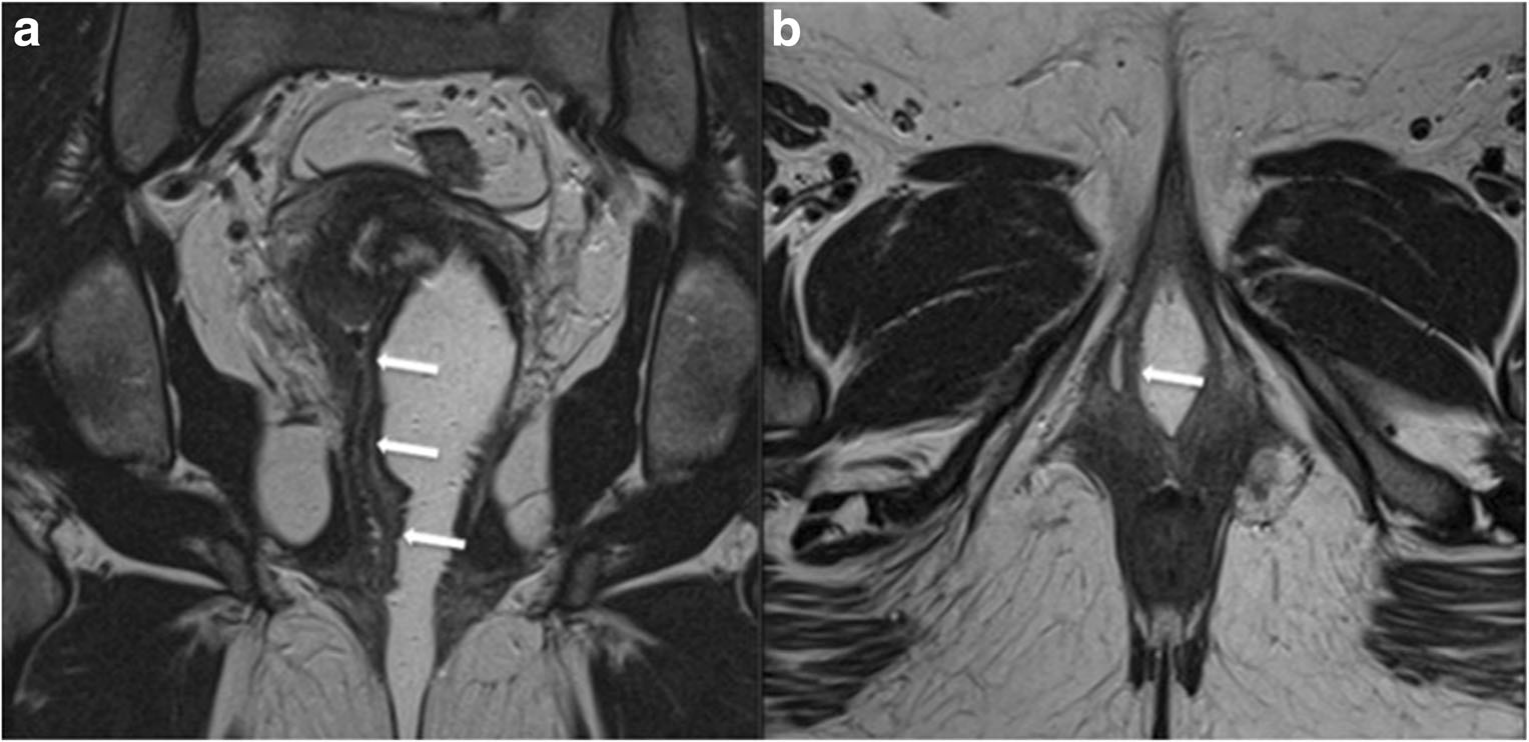
Vaginal septum revealed by MRI. T2W TSE coronal (**a**) and axial (**b**) images show a longitudinal non-obstructing vaginal septum (arrows), reaching the vaginal introitus, in keeping with a complete septum. The left vaginal canal is well distended with gel, while the right one has only a small amount, remaining collapsed. Of note, this septum was initially missed at gynaecologic examination and was unsuspected until the MRI examination

The use of intracavitary gel facilitates identification and characterisation of a vaginal septum, both transverse and longitudinal, on T2W sequences (Fig. 3) [14]. In addition, it helps to evaluate partial vaginal agenesis or segmental vaginal atresia before the surgical treatment [15].

The visualisation of vaginal/vulvar cysts is simplified and that of cervix/double cervix may also be improved (Fig. 2b) [15].

Vaginal filling is inexpensive and well tolerated [16]. Self-application of gel by the patient, when feasible, is a good strategy to overcome religious and personal issues, regarding vaginal manipulation. Additionally, it has the advantage not to overload the radiology staff when availability of time is limited.

A consensus was not reached among the respondents regarding vaginal gel use.

### MRI protocol

**Fig. 4:**
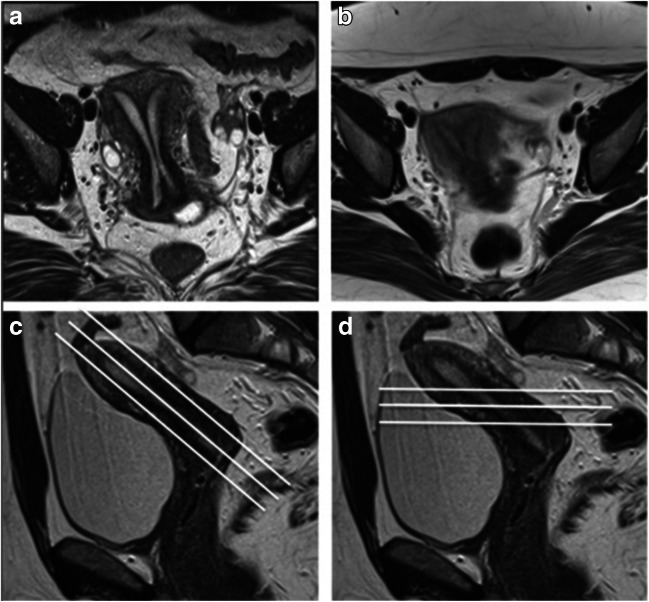
Importance of a dedicated MRI protocol. The same patient and the same MRI scanner but two examinations a few days apart. The patient was recalled to perform vaginal filling with US gel, for better characterisation of a vaginal septum. Axial T2W TSE images (**a**, **b**). The superior quality of image **a** is obvious, due to slice thickness (3 mm versus 5 mm), small FOV and prescribing the appropriate orientation. Image **a** depicts properly the uterine fundus and septum, important features for characterisation and classification of this uterine malformation (complete septate uterus). **c**, **d** Planning the acquisition from the sagittal sequence: axial oblique acquisition oriented by uterus long axis (**c**) yielding to a true coronal acquisition of the uterus shown in **a** versus an axial strict acquisition of the pelvis (**d**) yielding image **b**

In the study of FGTCA, the MRI protocol is based on the use of multiplanar 2D T2W high-resolution TSE/FSE sequences. T2W images require a slice thickness ≤ 4 mm with high matrix (512 × 512) and a small FOV to obtain a higher spatial resolution (Fig. 4a, b). Fat suppression is not recommended, due to the inherent contrast between the signal intensity of the uterus and the surrounding fat.

Multiplanar imaging is mandatory, and dedicated planes should be obtained along the long and short axis of the uterine body (yielding true coronal and true axial images of the uterus) in order to evaluate the external fundal contour and the cavity shape. For the purpose of uterine malformation classification, imaging the uterus in its true coronal plane is the most critical (Fig. 4) [4].

An oblique sequence obtained perpendicular to the cervical canal results in a short-axis view and allows accurate assessment of the cervix, i.e. the diagnosis of duplication or septation [17].

T1W FS sequences are required to detect blood products, in particular to evaluate the presence of haematometra, haematocolpos or endometriosis [5].

**Fig. 5:**
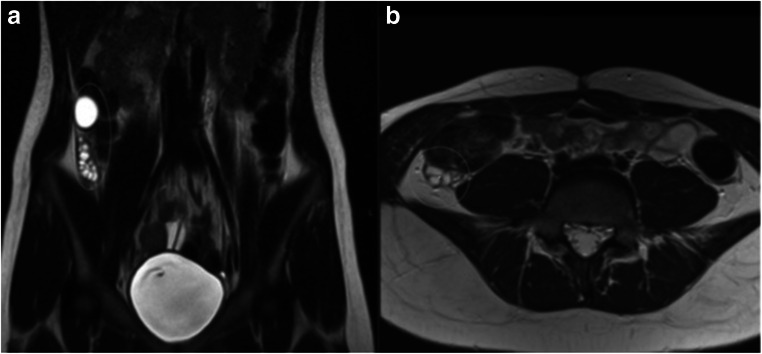
Unilateral ovarian maldescent in MRKH syndrome. T2 HASTE coronal (**a**) and axial (**b**) images depict the right ovary in an ectopic location, in the right paracolic gutter. Of note, the right ovary was reported as absent in a previous MRI examination that did not include a large FOV sequence, highlighting the importance of a dedicated MRI protocol

A coronal T2W sequence with a large FOV (such as SSFSE) should be acquired to evaluate the presence, position and morphology of the kidneys [18]. Additionally, this sequence can detect ectopic ovaries, which sometimes lie above the pelvic brim, outside the standard field for pelvic assessment, and may be reported as absent (Fig. 5) [19].

Gadolinium-based contrast agent is generally selectively used to assess incidentally detected coexisting conditions [12]. Delayed contrast-enhanced imaging may be useful if anomalous ureteral insertion is suspected [4].

In cases where a complex renal malformation is suspected, it might be useful to complete the examination performing MR-urography sequences.

Newer 3D T2W sequences seem to provide superior image quality and better 3D reconstructions compared with the classic 2D T2W sequences [20] with significant reduction in total scan time, compared with multiple 2D acquisitions [21]. The precise planning of the conventional scanning planes is not required [4] as images can be reformatted in any plane, including curved MPR [22] and double oblique through the uterus [18]. These 3D T2W sequences have different trade names: CUBE (GE), VISTA (Philips), SPACE (Siemens), among others [23].

Several MRI protocols for assessment of FGTCA are described in the literature. An abbreviated MRI protocol consisting of 3D T2W and non-enhanced axial T1W sequences for the pelvis and coronal SSFSE T2W sequences for the abdomen was recently proposed. This short protocol would provide useful information with regard to the uterine structure, the presence of blood products in the uterine cavity and the presence of renal anomalies [24].

The proposed ESUR-MRI protocol intends a dedicated evaluation of FGTCA (Table 4). Consensus was reached among the respondents.

**Table 4:**
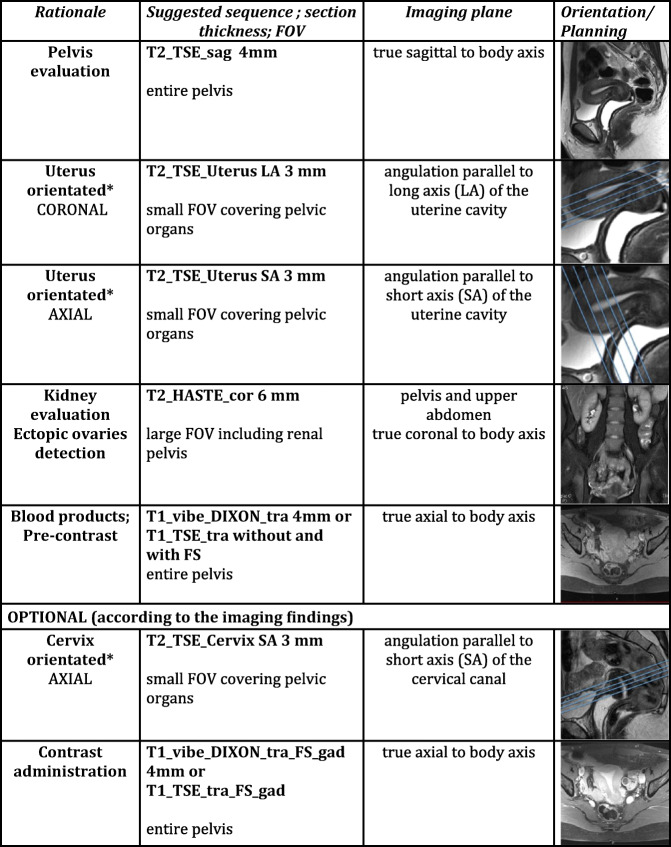
Proposed MRI protocol: sequences and rationale, with examples of how to plan uterus-orientated sequences

3D T2W may be an option to replace multiplanar T2W (*)

As the uterus can assume a multiplicity of positions in the pelvis, the planning of the uterus-orientated sequences needs to be tailored for each patient

### Value of MRI in the evaluation of FGTCA, associated anomalies and complications

#### Uterus, cervix and vagina

MRI provides detailed information on the uterovaginal anatomy, particularly in the study of the external profile of the uterine fundus and the cavity shape, and it also allows tissue characterisation of the possible septa, thus providing a complete classification of the specific anomaly [22, 25]. Currently, MRI presents the highest diagnostic accuracy in the characterisation of uterine anomalies (nearly 100%), owing to an excellent soft tissue resolution and multiplanar capability [26–28].

MRI is more accurate than US in the detection of rudimentary horns and can discriminate a non-functional rudimentary horn from a functional non-communicating rudimentary horn, thanks to its ability to evaluate the zonal anatomy and signal intensity involving the endometrial cavity and the possible presence of haematometra [22, 29].

#### Kidney and ureter

**Fig. 6:**
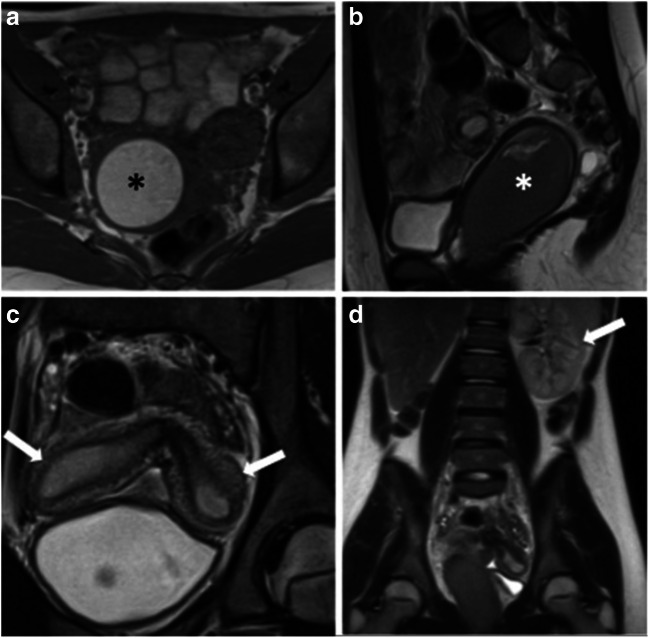
Herlyn-Werner-Wunderlich syndrome, a rare congenital anomaly of the urogenital tract, consisting in a triad of uterus didelphys, obstructed hemivagina (by a longitudinal vaginal septum attaching to the vaginal side wall) and ipsilateral renal agenesis. T1W TSE axial (**a**) and T2W TSE sagittal (**b**) images showing marked dilated right hemivagina with high T1 SI and low T2 SI contents, due to blood products, corresponding to haematocolpos (asterisk). Coronal T2W TSE image (**c**) depicts widely divergent uterine horns (arrows) of a didelphys uterus. T2 HASTE coronal image (**d**) with large FOV allows for renal evaluation, showing renal agenesis ipsilateral to the obstructed hemivagina. A left kidney with compensatory hypertrophy is depicted (arrow)

Uterine anomalies are often associated with urinary tract malformations, as the embryological development of the urinary system is closely associated with that of the genital tract (Fig. 6) [2]. Retrospective studies have demonstrated associated urinary tract anomalies in 17–42% of patients, with unilateral renal agenesis being the most common. Additional renal anomalies include multicystic dysplastic kidney, pelvic kidney, duplex kidney, horseshoe kidney and renal malrotation [30].

Patients with unilateral renal agenesis may have lower ureteric remnants inserting ectopically into the vagina, cervix and perineum, and distended with blood. Ectopic ureters have also been reported, with an apparently higher incidence compared with the general population [7]. Accurate knowledge of the ureteric course, presence of ectopic ureter or ureteric remnant, may be important preoperative information [7].

#### Ovaries

Ovarian maldescent is defined as lack of normal descent of the ovary during the embryonic development. Ovaries may be found in an ectopic position along the migration pathway from the lumbar region to the ovarian fossa [31].

The incidence of ovarian maldescent is increased in patients with MDA (17%) compared with women with normal uterine morphology (3%) [32].

MRI is considered the best imaging technique in the evaluation of abnormally located ovaries [33, 34]. The limited FOV of transvaginal US can preclude their identification [32].

#### Musculoskeletal anomalies

Fusion of vertebral bodies, hemivertebra, and malformation of the coccyx and sacral bone, scoliosis, have been reported in Mayer-Rokitansky-Küster-Hauser (MRKH) syndrome [35, 36]. These anomalies can be evaluated within the imaged FOV of MRI examination.

#### Endometriosis and pelvic adhesions

Endometriosis and pelvic adhesions are potential long-term complications of obstructive anomalies, due to retrograde menstrual flow from the obstructed side. MRI can detect these complications [4, 37, 38].

### Classification systems

Several classification systems exist for FGTCA; none of which is currently universally accepted, since all of them present some shortcomings.

In the medical literature, the ASRM classification is most widely used. In our group, the survey showed a slight preference for the ASRM classification over ESHRE/ESGE classification, probably reflecting that the ASRM classification was introduced earlier.

ASRM classification [39] distinguishes 7 different classes considering the Müllerian development and the relationship of the malformations to fertility [1, 4]. It is based mainly on uterine malformations while malformations of the vagina, adnexa or other associated malformations of non-Müllerian origin are not taken into account [40]. Furthermore, more complex type of malformations or obstructive anomalies as a result of cervical and/or vaginal aplasia/dysplasia in the presence of a functional uterus is not considered [41].

The vagina cervix uterus adnexa and associated malformation (VCUAM) classification [40] and the Embryological-Clinical classification proposed by Acien and Acien [1] tried to overcome these limitations, but failed to gain widespread acceptance, probably due to its complexity.

In 2013, the ESHRE/ESGE classification was proposed, based on anatomical considerations (Supplementary Material Fig. 1). Congenital anomalies are classified into 6 main classes, expressing uterine anatomical deviations deriving from the same embryological origin. Cervical and vaginal anomalies are classified in independent supplementary sub-classes. A non-Müllerian duct anomaly, imperforate hymen, is included in sub-class V3, along with transverse vaginal septum [8].

While this most recent classification system has overcome some problems associated with previous systems [41], it was shown in a comparison of the ESHRE-ESGE classification with the ASRM classification using 3D US [42] that the application of the ESHRE-ESGE criteria resulted in a relative overdiagnosis of septate uteri and insufficient inter- and intra-rater reliability [43], which potentially might lead to unnecessary surgical overtreatment.

### MR reporting of female genital tract congenital anomalies

The imaging approach can start with assessment of the uterus, cervix and vagina and ovarian location, followed by the evaluation of the kidneys and assessment of complications. Finally, a FGTCA classification system, ideally the same system used by the gynaecologist/surgeon at that institution, should be applied enabling efficient interdisciplinary communication.

A reporting checklist which highlights commonly overlooked imaging features is provided (Table 5).

**Table 5:** Tips for MRI interpretation and report, including imaging diagnostic issues and checklist according to the specific malformation, complications and associated anomalies

**Mayer-Rokitansky-Küster-Hauser syndrome**	
• Uterine remnants (uni- or bilateral) of small size are often missed at MRI	
• Report the presence/absence of endometrium in the rudimentary horn(s) or hypoplastic uterus	
• Report the vagina length (measurement in the sagittal plane)	
• Ovaries are commonly in an ectopic location in the abdomen • Look for skeletal malformations	
**Unicornuate uterus**	
• A small rudimentary horn is often missed or misinterpreted	
• Assess if the rudimentary horn is communicating or not communicating	
• Report the presence/absence of endometrium in the rudimentary horn	
• Describe the extension of attachment between rudimentary horn and hemi-uterus: separate/connected by a fibrous band/fused	
• Notice the topographic relationship between the rudimentary horn and the ipsilateral ureter—relevant for surgical planning	
**Septate uterus**	
• Report septum length and thickness	
• Report septum composition: fibrous/muscular/muscular + fibrous	
**Vaginal septum characterisation**	
• Transverse: low, middle, high	
• Longitudinal: obstructing, non-obstructing	
• Length, thickness	
• Presence/absence of fenestrations	
**Ovarian maldescent**	
• Diagnostic criteria: the upper pole of the ovary being above the pelvic brim, as defined by the pubic symphysis sacral promontory line; the upper pole of the ovary at or above the iliac artery bifurcation	
• May occur uni- or bilaterally	
• When an ovary is not in its normal location, seek it above the pelvic brim. The paracolic gutters are a common location. Very rarely the ovaries can be located in the inguinal canal	
**Kidneys and ureter**	
• Assessment of renal abnormalities: renal agenesis, pelvic kidney	
• Search for ectopic ureter	
• Search for ureteric remnant in patients with renal agenesis	
**Complications associated with obstructive anomalies**	
• Acute: haematocolpos, haematometra, haematometrocolpos, haematosalpinx, pyohaematocolpos, pyometra, pyosalpinx	
• Long term: endometriosis, pelvic adhesions	

## Conclusion

MRI is a ‘one-stop-shop’ imaging technique for a comprehensive evaluation of FGTCA, encompassing detailed uterovaginal evaluation, identification of abnormally located ovaries, associated anomalies and complications. MRI can also evaluate coexisting pelvic findings, such as leiomyomas, adenomyosis and tubal and ovarian pathologies. Finally, MRI is a non-invasive imaging modality, making it particularly useful in paediatric and virgo intacta patients.

Proper patient preparation and MR protocol are important to provide optimal examinations.

MRI of FGTCA encompasses a variety of imaging findings and the use of classification systems and structured reports can help in efficient transmission of information.

## Electronic supplementary material

ESM 1 (DOCX 1.87 mb)

## Abbreviations

ASRM: American Society for Reproductive Medicine
CT: Computed tomography
ESHRE/ESGE: European Society of Human Reproduction and Embryology/European Society for Gynaecological Endoscopy
ESUR: European Society of Urogenital Radiology
FGTCA: Female genital tract congenital anomalies
FPI-WG: Female Pelvic Imaging Working Group
FOV: Field of view
FS: Fat suppression
MPR: Multiplanar reformation
MRI: Magnetic resonance imaging
MRKH: Mayer-Rokitansky-Küster-Hauser
SI: Signal intensity
T1W: T1-weighted
T2W: T2-weighted
US: Ultrasound
VCUAM: Vagina cervix uterus adnexa and associated malformation
