# Supplementary material for: MRI of female genital tract congenital anomalies: European Society of Urogenital Radiology (ESUR) guidelines
Source: Eur Radiol. 2020 Mar 27;30(8):4272–83. doi: 10.1007/s00330-020-06750-8 (PMC7338830; doi:10.1007/s00330-020-06750-8)

**Supplementary Materials**

**Supplementary Table 1** Use of FGTCA classification systems in order of frequency

| ASRM (American Society for Reproductive Medicine) | n=5/17 |
| --- | --- |
| ESHRE/ESGE (European Society of Human Reproduction and Embryology/European Society for Gynaecological Endoscopy) | n=4/17 |
| ASRM + ESHRE/ESGE in the same report | n=4/17 |
| VCUAM (Vagina Cervix Uterus Adnexa and Associated Malformations) | n=2/17 |
| No classification system | n=2/17 |
| The Embryological-Clinical classification | n=0 |

**Supplementary** **Figure 1** ESHRE/ESGE classification of female genital tract congenital anomalies. (**a**) Schematic representation of the uterine anomalies. Class U2: internal indentation >50% of the uterine wall thickness and external contour straight or with indentation <50%, Class U3: external indentation >50% of the uterine wall thickness, Class U3b: width of the fundal indentation at the midline >150% of the uterine wall thickness. (**b**) Detailed classification of the uterine, cervical and vaginal anomalies. (Reprint from Grimbizis GF, Gordts S, Di Spiezio Sardo A et al (2013) The ESHRE/ESGE consensus on the classification of female genital tract congenital anomalies. Hum Reprod 28:2032–2044**)**


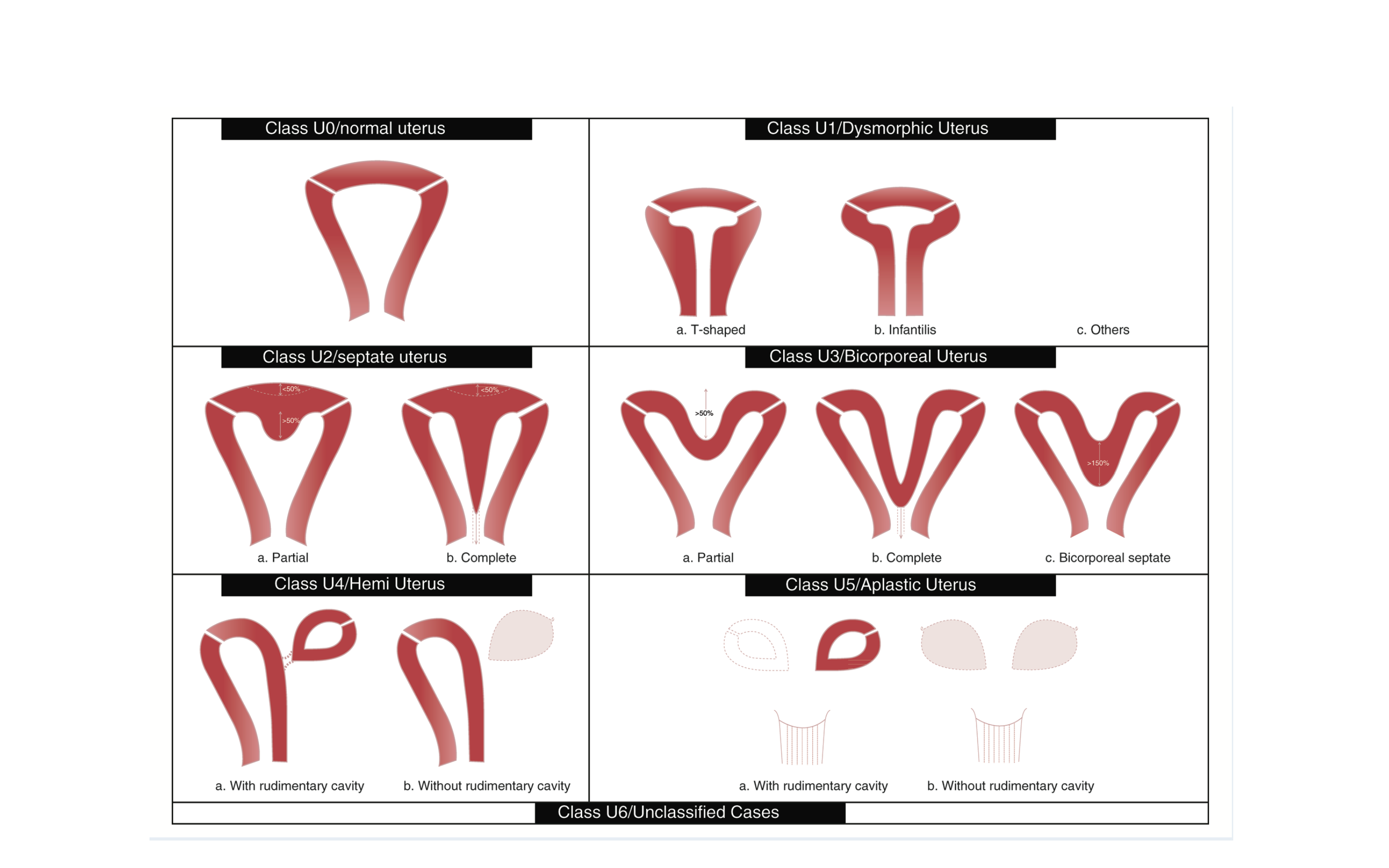


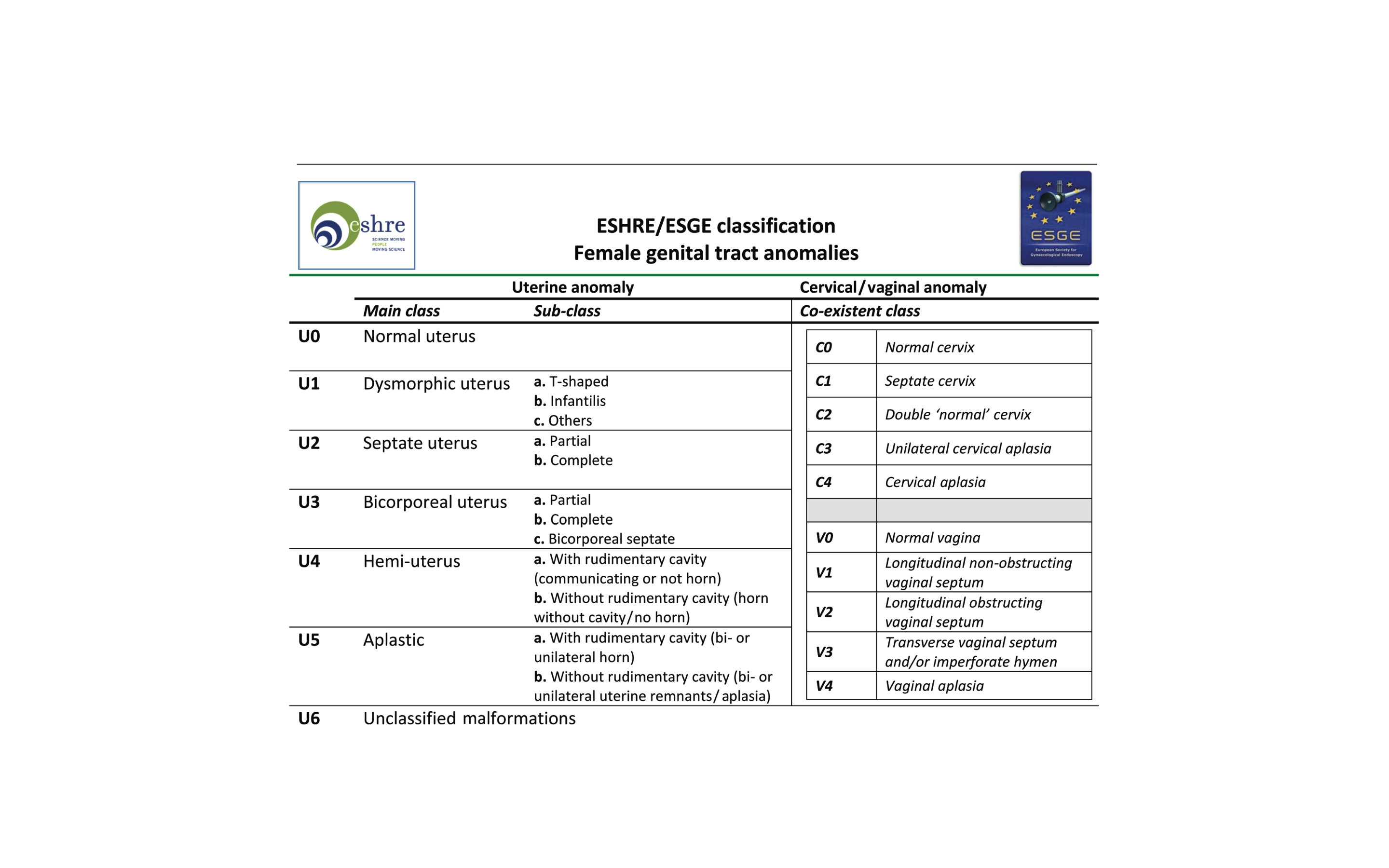

Supplement: Supplementary file 1 — (DOCX 1.87 mb) [file 330_2020_6750_MOESM1_ESM.docx]
